# Regulating cell fate of human amnion epithelial cells using natural compounds: an example of enhanced neural and pigment differentiation by 3,4,5-tri-*O*-caffeoylquinic acid

**DOI:** 10.1186/s12964-020-00697-5

**Published:** 2021-02-24

**Authors:** Meriem Bejaoui, Farhana Ferdousi, Yun-Wen Zheng, Tatsuya Oda, Hiroko Isoda

**Affiliations:** 1 Alliance for Research On the Mediterranean and North Africa (ARENA), University of Tsukuba, Tsukuba, Japan; 2 AIST-University of Tsukuba Open Innovation Laboratory for Food and Medicinal Resource Engineering (FoodMed-OIL), AIST, University of Tsukuba, Tsukuba, Japan; 3 Faculty of Life and Environmental Sciences, University of Tsukuba, 1-1-1 Tennodai, Tsukuba, Ibaraki 305-8572 Japan; 4 Department of Gastrointestinal and Hepato-Biliary-Pancreatic Surgery, Faculty of Medicine, University of Tsukuba, Tsukuba, Japan

**Keywords:** 3,4,5-tri-O-caffeoylquinic acid (TCQA), Human amniotic epithelial stem cells (hAECs), Cell cycle arrest, Differentiation, Pigment cell, Neurogenesis, Inflammation

## Abstract

Over the past years, Human Amnion Epithelial Cells (hAECs), a placental stem cell, are gaining higher attention from the scientific community as they showed several advantages over other types of stem cells, including availability, easy accessibility, reduced rejection rate, non-tumorigenicity, and minimal legal constraint. Recently, natural compounds are used to stimulate stem cell differentiation and proliferation and to enhance their disease-treating potential. A polyphenolic compound 3,4,5-Tri-*O*-Caffeoylquinic Acid (TCQA) has been previously reported to induce human neural stem cell differentiation and may affect melanocyte stem cell differentiation as well. In this study, TCQA was tested on 3D cultured hAECs after seven days of treatment, and then, microarray gene expression profiling was conducted of TCQA-treated and untreated control cells on day 0 and day 7. Analyses revealed that TCQA treatment significantly enriched pigment and neural cells sets; besides, genes linked with neurogenesis, oxidation–reduction process, epidermal development, and metabolism were positively regulated. Interestingly, TCQA stimulated cell cycle arrest-related pathways and differentiation signaling. On the other hand, TCQA decreased interleukins and cytokines expression and this due to its anti-inflammatory properties as a polyphenolic compound. Results were validated to highlight the main activities of TCQA on hAECs, including differentiation, cell cycle arrest, and anti-inflammatory. This study highlights the important role of hAECs in regenerative medicine and the use of natural compounds to regulate their fate.

**Video abstract**

**Video abstract**

## Introduction

Finding a reliable, effective, and safe source of stem cells to treat various incurable diseases constitute one of the most important challenges of regenerative medicine in this modern society [1]. Broadly, stem cells are classified into embryonic and adult stem cells. Embryonic Stem Cells (ESCs) can differentiate into various types of precursor cells for the regeneration of damaged tissue, as for adult stem cells like Mesenchymal Stem Cells (MSCs), they are widely used in repairing and maintaining the tissue that were isolated from [2]. However, both sources of stem cells have limitations in clinical practice, present ethical and legal issues, spontaneous differentiation, difficulty of extraction, and tumorigenicity [3, 4].

In recent years, attention was given to cells derived from discarded placenta, as they are emerging as a novel alternate source of pluripotent and multipotent stem cells. The amniotic membrane, which forms the innermost layer of the fetal membrane, is of particular interest. Two types of stem cells can be derived from the human amniotic membrane: the Human Amniotic Mesenchymal Stromal Cells (hAMSCs) and the Human Amniotic Epithelial Cells (hAECs) [5, 6]. The hAECs have been reported to have properties similar to both ESCs and MSCs because of their ability to differentiate into verious types of cells generated from all three germ layers, and to expand in culture [7, 8]. Most importantly and in contrast to ESCs, and MSCs, hAECs do not form teratomas in vivo, have low immunogenicity, are non-tumorigenic when transplanted, are obtained with non-invasive procedure, and have minimal ethical and legal barriers [1]. Additionally, these cells are gaining a huge interest among the stem cell research community because they are able to differentiate into a broad spectrum of cell types such as neuronal, epithelial, adipocyte, pancreatic, cardiac, osteogenic, and hepatic cells [9–11]. Moreover, hAECs have anti-inflammatory, wound healing, and immunomodulatory properties, as well as promoting re-epithelization and inhibiting angiogenesis. These beneficial activities of hAECs have contributed to their potential use in clinical trials [12, 13].

Currently, substances like synthetic cytokines and proteins and recombinant growth factors are used to induce stem cell differentiation. However, several side effects were observed including, toxicity, malignancy, and a high risk of rejection. Moreover, these molecules degrade rapidly and need constant replacement, which makes the process costly and limits the use [14, 15]. In this context, finding new molecules to act on stem cell fate has become one of the biggest challenges within the scientific community. Recently, the use of plant-based natural products that may affect the differentiation of stem cells has been approved in the mainstream of medicine because of their high availability, minimum toxicity, and low secondary effects [16, 17]. It has been reported that different natural compounds may regulate early biological events in hAECs to induce directed differentiation such as rosmarinic acid induced neuronal differentiation, whereas isorhamnetin induced hepatic-lineage specific differentiation in hAECs [18, 19]. Also, verbenalin-treated hAECs revealed a therapeutic potential for Alzheimer’s disease [20].

Caffeoylquinic Acid (CQA) and its derivatives are polyphenolic compounds found in a variety of plants and exhibited several activities including antioxidant, antibacterial, anticancer, antihistaminic, and pigmentation-regulating effects [21–27].We distinguish as one of CQA derivatives, 3,4,5-Tri-*O*-Caffeoylquinic Acid (TCQA) with an IUPAC name (3R,5R)-3,4,5-tris[[(E)-3-(3,4-dihydroxyphenyl)prop-2-enoyl]oxy]-1-hydroxycyclohexane-1-carboxylic. Previously, this compound was reported to inhibit aldose reductase, and to prevent hypertension, hyperglycemia, and Alzheimer's disease [28]. TCQA was found to enhance Adenosine Triphosphate (ATP) production in human neuroblastoma SH-SY5Y cell, and to improve deficit of learning and memory in the aging model Senescence-Accelerated Prone Eight (SAMP8) mice [29, 30]. Moreover, in our previous studies, we demonstrated that TCQA promoted hair regrowth and pigmentation in eight-weeks-old C3H male, human dermal papilla cells and human melanocytes via the upregulation of Wnt/β-catenin pathway and its target genes. TCQA upregulated pathways involved in skin and hair stem cell differentiation and the protein expression of CD34, known to be enhanced when melanocytes stem cells are differentiating [31–33]. On the other hand, TCQA triggered the differentiation of Human Neural Stem Cells (hNSC) via Bone Morphogenetic Protein (BMP) pathway and the induction of cell cycle arrest [27]. Interestingly, the melanocytes are originally from the neural crest cells before they migrate and colonize the skin and the hair follicle. Their differentiation is induced by various pathways, including Notch, BMP, and Wnt/β-catenin pathways, which have been reported to be regulated by TCQA in vivo and in vitro [33, 34].

Taken together, TCQA is a potential candidate to induce stem cell differentiation into neuronal and pigment cell lineage. In this current research, the effect of TCQA on the activation of hAECs differentiation was investigated. Global gene expression profiling using DNA microarray showed the upregulation of genes significant in pigment and neuronal cell differentiation, neurogenesis, ATP process, inflammation, and cell cycle arrest.

## Materials and methods

### TCQA preparation

For the in vitro assays, synthesized TCQA (97% pure) was used. The stock solution was prepared in 70% ethanol and then TCQA was diluted in purified water and in the appropriate cell culture medium before adding to the cells. In this study, this compound was kindly provided by Dr. Kozo Sato from Synthetic Organic Chemistry Laboratories, the FUJIFILM Corporation (Kanagawa, Japan).

### Isolation of hAECs

The amniotic samples from healthy human donors were collected during full-term C-sections. Then, an aseptic separation between the amnion and the chorion was assessed, and washing with 200 ml Hank’s Basic Salt Solution Calcium and Magnesium Free (CMF-HBSS) to eliminate blood clots was conducted. The membrane was cut into small parts using sterile surgical scissors and placed then in 50 ml tubes where a pre-digestion buffer (CMF-HBSS, 0.5 mM Ethylene Glycol-bis β-Aminoethyl Ether (EGTA), 20 ml) was added under a slight agitation for 30 s. The previous solution was discarded and the different parts of the amnion were transferred to new 50 tubes containing 20 ml of the pre-digestion buffer following by 10 min incubation at 37 °C. After removing the pre-digestion buffer, a solution of 20 ml of 0.05% trypsin–EDTA was added to the tissue for 40 min at 37 °C and then the tubes were placed on ice. The culture medium Dulbecco’s Modified Eagle Medium (DMEM) (Gibco, Karlsruhe, Germany) supplemented with 10% Fetal Bovine Serum (FBS) (Gibco, Karlsruhe, Germany) and 0.1% Penicillin/Streptomycin (Gibco, Karlsruhe, Germany) was added to the trypsin digest and centrifuged at 200 g for 10 min at 4 °C. The pellet was resuspended in 10 ml of the medium and then filtered with a 100 μm filter (BD Falcon, England, UK) to eliminate the tissue aggregates. The cell suspension containing the amnion epithelial cells was collected and the membrane was discarded.

### Cell culture

HAECs were extracted then maintained in Placenta Epithelial Cell Basal Medium (PromoCell, Heidelberg, Germany). The medium was changed every other day and the cells were constantly monitored to detect if any change occurs in the morphology or structure. The cells were cultured in suspension and for subculture, the cells were washed twice with 10 ml Phosphate Buffered Saline (PBS), then 3 ml of pre-warmed pre-digestion buffer was added to the cells for 5 min at 37 °C. The cells were trypsinized for 10 min prior to adding 5 ml of DMEM, then centrifuged at 200 rpm for 4 min at 4 °C. The supernatant was discarded and the pellet was resuspended in the culture medium and centrifuged again under the same conditions. The supernatant was removed and the cells were resuspended in the placental cell medium before seeding in Petri dishes.

### 3D spheroid culture

The hAECs were cultured in 3D system. Firstly, a solution of 50 mg Lipidure TM (NOF Corporation, Tokyo, Japan) dissolved in 10 ml 100% ethanol was added to the 3D culture plate (Kuraray Co, Tokyo, Japan) for 2 min. After removing the solution and a 3 h drying, 400 μl PBS was placed in each well and the plate was centrifuged for 15 min at Room Temperature (RT), and checked under the microscope to ensure that there was no bubble formation. The PBS was then discarded and a washing with new PBS solution was conducted twice and the plates were then ready for 3D culture.

The cells were seeded at a density of 1 × 10^6^ in Placenta Basal Epithelial Cell Medium into each well of the previously prepared culture plates to ensure the spheroids formation. Day 0 control (D0 control) samples were collected before adding the treatment to the cells. The cells were maintained for one-week culture with changing the medium with 0 and 20 µM TCQA every 48 h. At the end of the week, we established two groups: the treatment (D7 TCQA) and control (D7 control).

### RNA extraction

The total RNA was extracted using ISOGEN kit (Nippon Gene, Tokyo, Japan) following the manufacturer’s instructions. The hAECs were cultured as previously described, then washed with cold PBS before the total RNA extraction. The total RNA was quantified using a NanoDrop 2000 spectrophotometer (NanoDrop Technologies, Massachusetts, USA).

### Microarray analysis

Microarray hybridization probes were generated from isolated RNA extracted from the hAECs treated with 0 or 20 µM TCQA using ISOGEN solution (Nippon Gene, Tokyo, Japan) following the manufacturer’s instructions. Briefly, the extracted RNA was amplified and biotin-labeled as aRNA. Then, the fragmented, biotin-labeled aRNA was hybridized to the Affymetrix Human Array strips (HG U219) containing probes for approximately 45,141 transcripts and variants. The chips were washed and stained in the Gene Atlas Fluidics Station 400 (Affymetrix, Santa Clara, USA) and the resulting image was scanned using the Gene Atlas Imaging Station (Affymetrix, Santa Clara, USA). The generated data were normalized using the Transcriptome Analysis Console (TAC) Software (version 4.0.1) following the Robust Multichip Average (RMA) algorithm. Gene expression values refer to Tukey's Bi-weight average of gene level intensity of all the replicates in a condition. Genes with a fold change ≥ 1.3 (in linear space) and *P*-value ≤ 0.05 (One-way between-subject ANOVA) were considered as differentially expressed genes (DEGs). Database for Annotation, Visualization, and Integrated Discovery (DAVID) bioinformatics resources 6.8 and Gene Set Enrichment Analysis (GSEA) were used for further Gene Ontology (GO) analysis of DEGs in each set of comparison [35–37]. GOs and pathways with p-value < 0.05 (modified Fisher’s Exact test with default EASE score threshold 0.1) were considered significant.

### Quantitative real-time PCR analysis

The hAECs were cultured, treated, and the total RNA was extracted as described above. The RNA was extracted from the three groups: D0 control, D7 control, and D7 TCQA. The cDNA was synthesized from the extracted RNAs using SuperScript IV reverse transcription kit (Invitrogen, CA, USA) with a cycling protocol as follows: 95 °C for 10 min, 40 cycles of 95 °C for 15 s, and 60 °C for 1 min. The real-Time PCR was performed using 7500 Fast Real-Time PCR Software 1.3.1 (Applied Biosystems, CA, USA) with TaqMan probes specific to *CTNNB1, BMP5,* Melanocortin 1 Receptor (*MC1R),* Dermokine *(DMKN),* Cyclin-Dependent Kinase Inhibitor 1 *(p21),* Versican (*VCAN),* Interleukin 6 *(IL6)*, and Tumor Necrosis Factor Alpha (*TNFa)* (Applied Biosystems, CA, USA). *GAPDH* (Applied Biosystems, CA, USA) was used as an endogenous control. In order to calculate the relative mRNA expression levels using the endogenous control, the 2 − ΔΔCt method was assessed.

### Statistical analysis

For the quantitative real-time PCR, all the experiments were performed three times and the results were expressed as mean of ± standard deviation (SD). The Student’s t-test was performed when two groups were compared. *P*-value of ≤ 0.05 was considered significant.

### Ethics approval

The protocol of extraction of hAECs was checked and approved by the Ethical Review Committee of the University of Tsukuba. Informed written consent was obtained from the mothers who donated the placenta after delivery.

## Results

### TCQA affected differentiation-associated genes in seven days treated hAECs compared with Day 0 control

**Fig. 1:**
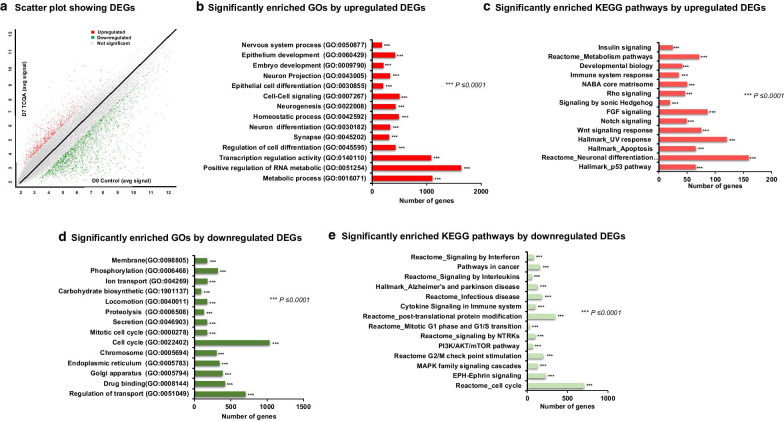
Microarray profiling of D7 TCQA-treated versus D0 control hAECs. **a** Scatter plot showing the DEGs. X-axis represents the average signal intensities (log2) in D0 control. Y-axis represents the average signal intensities (log2) in D7 TCQA-treated hAECs. The red color represents the upregulated DEGs, green color represents the downregulated DEGs, and grey color represents the nonsignificant genes. **b** Significantly enriched GOs by upregulated (analyzed using GSEA). **c** Significantly enriched KEGG pathways by upregulated DEGs (analyzed using DAVID and GSEA). **d** Significantly enriched GOs by downregulated (analyzed using GSEA). **e** Top significantly enriched KEGG pathways by the downregulated DEGs (analyzed using DAVID and GSEA). Each bar is arranged according to significance (p-values) and represents the number of DEGs

The RNA was collected from hAECs at Day 0 prior to treatment and seven days after treatment with 20 µM TCQA, then global gene expression analysis was conducted on two biological replicates (D7 TCQA-treated and D0 control hAECs). Results revealed that 3119 genes were differentially modulated by TCQA, out of which, 707 were upregulated while 2421 downregulated (Fig. 1a).

Figure 1b illustrated the GOs of the upregulated gene after seven days of treatment compared with D0 control. TCQA significantly enhanced genes associated with metabolic process (GO: 0,016,071), neuron differentiation (GO: 0,030,182), epithelial cell differentiation (GO: 0,030,855), regulation of cell differentiation (GO: 0,045,595), and nervous system process (GO: 0,050,877). Analysis of the upregulated pathways revealed that the affected signaling are Wnt/ β-catenin, Fibroblast Growth Factor (FGF), Notch, Sonic Hedgehog (Shh), and neural differentiation-related pathways (Fig. 1c).

On the other hand, TCQA negatively modulated genes significant for the regulation of the cell cycle (GO: 0,022,402), mitotic cell division (GO: 0,000,278), and drug binding (GO: 0,008,144) (Fig. 1d). For the downregulated pathways, we observed the regulation of Mitogen-Activated Protein Kinases (MAPK) pathway, PI3K/AKT/mTOR, cytokine and interferon signaling, neurodegenerative diseases, and cell cycle progression and transition-related pathways (Fig. 1e). These results indicated that seven days of treatment with TCQA compared with D0 control, positively regulated neurogenesis and induced epithelial and neuronal cell differentiation, while repressing genes linked with inflammation and cell cycle progress and division, which favorite differentiation over proliferation in hAECs.

**Fig. 2:**
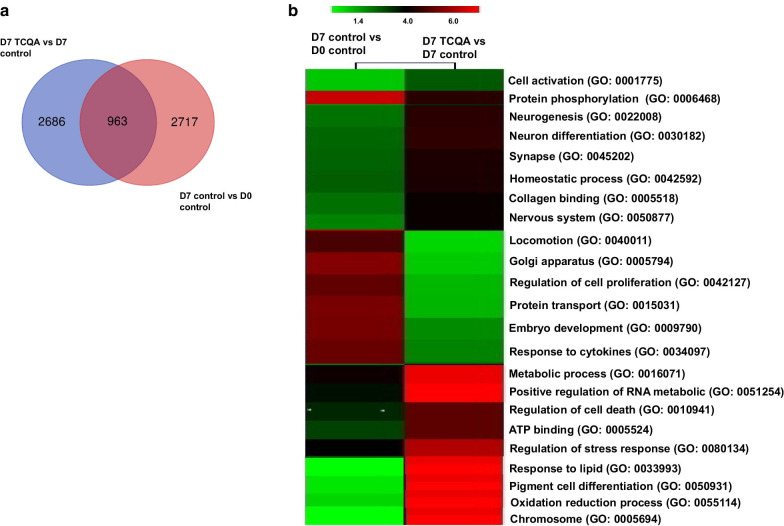
Comparison of gene profiling between D7 TCQA-treated versus D7 control hAECs with D7 control versus D0 control. **a** Venn diagram showing common and unique sets of DEGs between each exposure. **b** Heat map showing the significance of biological processes in two comparison sets—D7 control versus D0 control and D7 TCQA-treated versus D7 control hAECs. Heat map was generated using Morpheus online software (https://software.broadinstitute.org/morpheus)

Interestingly, we compared D7 TCQA versus D7 control with D7 control versus D0 control to observe the fate of hAECs without treatment. A Venn diagram was created to compare the DEGs between the two sets of comparisons (Fig. 2a). Clustering analysis was then performed and the upregulated genes by D7 TCQA versus D7 control comparing with D7 control versus D0 control were linked to cell differentiation, neurogenesis, cell cycle arrest, and pigment cell differentiation. The comparison of the downregulated genes showed the modulation of genes related to cell activation, cell cycle, and regulation of cell proliferation (Fig. 2b).

Moreover, a comparison between D7 TCQA versus D0 control with D7 TCQA versus D7 control was performed and the results showed as well an upregulation in differentiation-related pathway upon TCQA treatment (Additional file 1: Suppl Fig. 1).

### TCQA enhanced neuronal and pigment cell differentiation-associated genes in seven days treated hAECs compared with Day 7 control

**Fig. 3:**
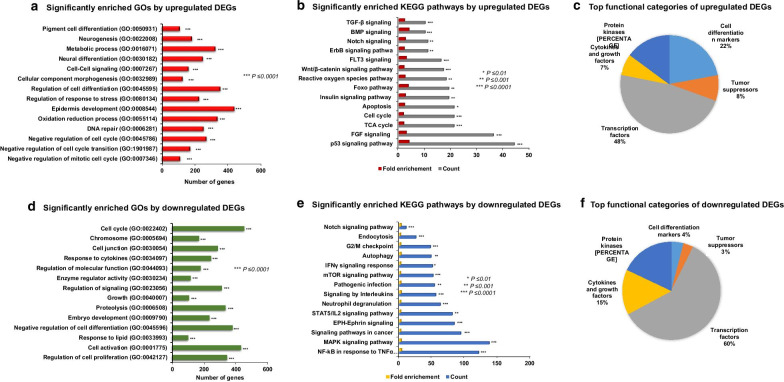
Gene expression profiling of D7 TCQA-treated versus D7 control hAECs. **a** Significantly enriched GOs by upregulated DEGs. **b** Significantly enriched KEGG pathways by upregulated DEGs. **c** Top functional categories of upregulated DEGs. **d** Significantly enriched GOs by downregulated DEGs. **e** Significantly enriched KEGG pathways by downregulated DEGs. **f** Top functional categories of downregulated DEGs

DNA-microarray analysis was assessed on two biological replicates (D7 TCQA-treated and D7 control hAECs). GSEA showed that several priori-defined gene sets associated with cell differentiation (GO: 0,045,595) including pigment cell (GO: 0,050,931) and neuron cells (GO: 0,030,182), epidermis development (GO: 0,008,544), negative regulation of cell cycle transition (GO: 1,901,987), DNA repair (GO: 0,006,281), and oxidation–reduction process (GO: 0,055,114) were significantly enriched by the DEGs between D7 TCQA and D7 control cells (Fig. 3a). Then to know more about the upregulated pathway, DAVID analysis was performed and results showed an upregulation of differentiation-related pathways such as Wnt/ β-catenin, BMP, Notch, ErbB, and Tumor Protein p53 (p53) (Fig. 3b). Additionally, TCQA affected Foxo pathway, insulin signaling, and Tricarboxylic Acid (TCA) cycle (Fig. 3b). The DEG were classified according to their function and 22% of the genes are classified as cell differentiation markers, 8% as tumors suppressors, and 48% as transcription factors (Fig. 3c).

The top 12 upregulated genes in D7 TCQA-treated hAECs were classified in Table 1. The most affected genes were associated with cell cycle arrest including *p21* with a fold-change of 4.71. Additionally, genes involved with pigment and neural cell differentiation like *CTNNB1*, *VCAN*, *BMP5, DMKN*, and *MC1R*, were upregulated up to 2.66, 2.27, 2.12, and 2.02 fold-change, respectively (Table 1). Putting these results together, TCQA stimulated the differentiation of hAECs towards neural and pigment cell lineage.

**Table 1:** Top upregulated genes in D7 TCQA-treated hAECs (vs D7 control) *

Gene symbol	Gene name	Biological function	Fold-change	*P* value**
*p21*	Cyclin-dependent kinase inhibitor 1	Tumor suppressor; Cell cycle arrest	4.71	0.005
*DYNC1H1*	dynein cytoplasmic 1 heavy chain 1 inhibitor 2	Tumor suppressor; Cell cycle arrest	2.74	0.028
*CTNNB1*	Catenin (cadherin associated protein), beta 1	Melanocytes stem cells differentiation; Neural differentiation; pigmentation	2.66	0.040
*TCF4*	Transcription factor 4	Canonical Wnt signaling pathway activation	2.48	0.005
*VCAN*	Versican	Intercellular signaling; Regulation of differentiation	2.27	0.031
*CDKN1*	Cyclin-dependent kinase inhibitor 1	Tumor suppressor; Cell cycle arrest	2.22	0.004
*BMP5*	Bone morphogenetic protein 5	Neural crest cell survival; dendritic growth; Keratinocyte and skin stem regulation	2.12	0.002
*DMKN*	Dermokine	Regulator of keratinocyte differentiation	2.07	0.002
*MC1R*	Melanocortin 1 receptor	Melanin biosynthetic process; pigmentation; pigment cell regulation	2.02	0.047
*GATA2*	GATA binding protein 2	Nervous system development; Neuron and dendritic cell differentiation; Wound healing	1.91	0.025
*NDUFS2*	NADH:ubiquinone oxidoreductase core subunit S2	Oxidation–reduction process, ATP synthesis	1.85	0.021
*FOXRED1*	FAD-dependent oxidoreductase domain containing 1	Oxidation–reduction process, ATP synthesis	1.77	0.001

*Genes functions were obtained from NCBI. **ANOVA was performed to assess the level of significance between groups

### TCQA downregulated cell cycle and inflammation-associated genes in seven days treated hAECs compared with day 7 control

Previously, we showed that TCQA downregulated cell cycle and inflammation-related genes compared with Day 0 control. This effect is confirmed as well comparing TCQA and Day 7 control as illustrated in Fig. 3d. Moreover, GO analysis showed a downregulation in the regulation of cell proliferation (GO: 0,042,127), cell activation (GO: 0,001,775), and negative regulation of cell differentiation (GO: 0,045,596) (Fig. 3d). KEGG pathway analysis showed the downregulation of G2/M checkpoint, interleukins, TNFα/ NF-κB signaling, and Inteferon Gamma (IFNγ) signaling (Fig. 3e). The classification of the genes according to their function revealed that TCQA negatively modulated 15% cytokines and growth factors, 4% cell differentiation markers, 3% tumors suppressors, and 60% transcription factors (Fig. 3f). Table 2 summarizes the top downregulated genes by TCQA upon seven days of treatment in hAECs. Inflammatory genes expression such as *TNFα, IL6*, and Interleukin 8 (*IL8)* was downregulated by TCQA up to a fold-change, respectively, − 3.65, − 3.32, and − 2.88 (Table 2).

**Table 2:** Top downregulated genes in D7 TCQA-treated hAECs (vs D7 control) *

Gene symbol	Gene name	Biological function	Fold-change	*P* value**
*TNFα*	Tumor necrosis factor alpha	Inflammatory response; Cell activation; Cell death; Negative regulation of apoptotic process	− 3.65	0.004
*IL6*	Interleukin 6	Negative regulation of collagen biosynthesis; Inflammatory response; Cytokine response	− 3.32	0.009
*IL8*	Interleukin 8	Pro-inflammatory response; Angiogenesis; Phagocytose	− 2.88	0.016
*FOS*	FBJ osteosarcoma oncogene	Cyclin D1 activation; Cell cycle regulation	− 2.80	0.001
*ABCG1*	ATP-binding cassette transporter G1	Cholesterol accumulation; T cell homeostasis alteration	− 2.65	0.045
*PTGS2*	Prostaglandin G/H synthase 2	Production of inflammatory prostaglandins; Cell adhesion; Resistance to apoptosis; Tumor angiogenesis	− 2.65	0.033
*SORT1*	Sortilin	Neuronal apoptosis: Coronary artery disease; Tumor cell survival	− 2.45	0.012
*GPNMB*	Glycoprotein non-metastatic melanoma protein B	Alzheimer disease marker; Regulator of melanoma tumor growth	− 2.43	0.004
*IRF2*	Interferon regulatory factor 2	Oncogene; Human leukaemia cell growth	− 2.24	0.001
*KLK6*	Kallikrein-6	Pathogenesis of Parkinson disease; Collagen and filament degradation	− 2.22	0.023
*TRPV3*	Transient receptor potential cation channel subfamily V member 3	Negative regulator of hair growth and cycling; Suppresses keratinocyte proliferation	− 2.17	0.043
*IFNLR1*	Interferon, lambda receptor 1	Inflammatory and cytokine response	− 2.12	0.002

*Genes functions were obtained from NCBI. **ANOVA was performed to assess the level of significance between groups

Hence, TCQA stimulated the differentiation of hAECs via decreasing the cell cycle activity and the cell proliferation rate. Moreover, it has an anti-inflammatory activity as it decreased cytokines and interleukins-related genes.

### TCQA regulated cell differentiation, inflammation, cell cycle, and ATP content in seven days treated hAECs

**Fig. 4:**
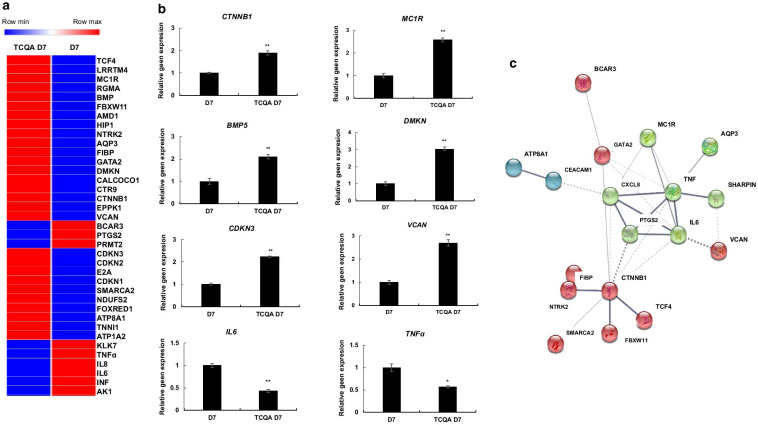
Molecular functions induced in D7 TCQA-treated compared with D7 control hAECs. **a** Heat map showing relative expression intensities of DEGs. Heat map was generated using Morpheus software. **b** Gene expression of *CTNNB1, MC1R, VCAN, DMKN, IL6*, and *TNFα* in D7 TCQA-treated and D7 control hAECs. The mRNA level was quantified using TaqMan real-time PCR. Results represent the mean ± SD of three independent experiments. The Student’s t-test was used to compare the mean values of two groups. *P ≤ 0.05; **P ≤ 0.01. **c** Possible protein interactions among the genes classified in the heat map. Figure was generated using an online software STRING (https://string-db.org/)

To understand more the effect of TCQA on hAECs, a heat map was created using Morpheus software. Results revealed that TCQA had five main functions: stimulation of neural and pigment cell differentiation, cell cycle arrest, tumor suppressor and apoptosis regulator, ATP content stimulator, and anti-inflammatory activity (Fig. 4a). In addition, the microarray results were further confirmed by RT-PCR that showed an enhancement of the gene expression of *CTNNB1, BMP5, VCAN, MC1R*, and *DMKN* known to be involved in neural and pigment cell differentiation process. Whereas, p21 gene expression was investigated because of its role in cell cycle arrest (Fig. 4b). On the other hand, the expression of *IL6* and *TNFα* was negatively regulated after seven days of treatment, further proving the anti-inflammatory effect of TCQA (Fig. 4b). To look for possible protein interaction between the genes previously grouped in the heat map, STRING software was used. Figure 4c represents the obtained results showing that there is an interaction between the DEG on the protein level.

## Discussion

Recently, scientists are looking for new sources of cells for regenerative medicine. Human amniotic membrane consists of an epithelial layer, a basal lamina, and an avascular mesenchymal layer [38]. The epithelial layer contains a high number of epithelial cells, which are gaining a great interest in cell-based therapies and considered to have the potential to overcome the existing challenges such as immunological rejection, tumorigenesis, and ethical problems [5, 39].

HAECs are multipotent cells because of their ability to differentiate into different lineages [40]. Plant extracts and their bioactive compounds have received considerable attention and been accepted in the mainstream of medicine, especially because of their protective properties and their potential to induce the proliferation and differentiation of stem cells [41]. In this current study, we showed the effect of the polyphenolic compound TCQA on inducing the differentiation of hAECs into neural and pigment cell lineage via the upregulation of differentiation- and cell cycle arrest-associated genes. Additionally, TCQA has been found to exhibit an anti-inflammatory and ATP stimulating effect.

In this study, the hAECs were obtained from Tsukuba Human Tissue Biobank Center (THB) established at Tsukuba University Hospital [42]. Primarily, from each placenta sample (donated by healthy mothers who underwent cesarean section), three sets of specimens were collected for the collection of placenta-derived somatic stem cells: umbilical cord for endothelial progenitor cells, Wharton’s jelly for mesenchymal stem cells, and amniotic membrane for amniotic epithelial stem cells. The primary amniotic epithelial cells were heterogeneous; however, the hAECs isolated from the adherent subpopulations of passaged primary cells could widely express stemness markers [43]. Besides, hAECs cultured in a 3D microenvironment as spheroids have highly expressed the stemness-related genes compared to their 2D counterpart as well as compared to iPSCs and MSCs [18].

Firstly, we compared D7 TCQA-treated hAECs with D0 untreated control hAECs. Our findings showed that TCQA significantly enriched neurogenesis-, nervous system process-, neuron and epithelial cell differentiation-associated genes (Fig. 1). Actually, several studies reported that hAECs express neural markers even before differentiation and they can synthesize and release dopamine, acetylcholine, noggin, catecholamines, and neurotrophic factors; and as mentioned previously, hAECs have a potential to differentiate into neural lineage upon rosmarinic acid treatment [13, 18, 44]. Various signaling pathways are engaged in the process of neural development and hNSC differentiation. Wnt signaling has been implicated in promoting self-renewal during neural development and it has been shown that this pathway is considered to be a cell–cell and a cell–matrix contact-regulated inducer of neurogenesis [45, 46]. FGF pathway has a key role in early neural differentiation and development, the acquisition of neural identity, and chromatin regulation [47–49]. Hence, signaling pathways control neuron fate and regulation, and this activity is achieved through their regulatory interactions to decrease or enhance an activity. The Wnt/β-catenin and FGF pathways regulate not only neuronal stem cell differentiation but also proliferation, and it is known that the crosstalk and the balance between them induce the growth and the development of adult brain, and the appropriate segregation of neuronal populations [50, 51]. Here, upon TCQA treatment, Wnt- and FGF-related genes were upregulated in seven days treated hAECs compared with D0 control. Interestingly, hAECs not only showed differentiation markers but also neurogenesis genes were significantly stimulated (Fig. 1b, c). This result can be explained by the upregulation of Notch pathway known to regulate neurogenesis and brain development positively at both embryonic and adult stages [52].

Results showed as well a downregulation in cell cycle and inflammation significant genes and pathways (Fig. 1d, e). In addition, we checked the effect of D7 TCQA with D7 control and we compared them with D7 control versus D0 control. TCQA treatment significantly regulated genes linked with cell cycle arrest, regulation of cell differentiation, and neurogenesis (Fig. 2). On the other hand, it downregulated genes and pathways involved with cell activation, cytokine response, and embryonic development (Fig. 2). Therefore, TCQA stimulated the differentiation of hAECs compared with the untreated cells. GSEA analysis of D7 TCQA versus D7 control, showed that TCQA enriched pigment cell differentiation, epidermis development, and oxidation–reduction sets and upregulated Wnt/β-catenin and ErbB signaling pathways (Fig. 3a, b). The Wnt/β-catenin signaling pathway plays an important role in the regulation of pigmentation, melanocyte development, and melanocyte stem cell differentiation [53, 54]. Previously, we reported the effect of TCQA on increasing the pigmentation in human melanocytes, in B16F10 cells, and in eight-weeks-old C3H male mice through the upregulation of β-catenin and its target genes and, to enhance the protein expression of CD34 known as a melanocytes stem cells differentiation marker [32]. The EdnrB pathway is implicated in melanocytes stem cell regeneration and differentiation via crosstalk with Wnt signaling [55].

The GO analysis revealed that TCQA positively stimulated neural cell differentiation, cell cycle arrest, and neurogenesis, compared with D7 control (Fig. 3). Actualy, no obvious morphological changes supporting neurological or pigment cell differentiation could be observed due to the spheroid structure of the cells. Moreover, the treatment duration was only seven days, which may be enough to induce early biological events for directed differentiation but is too early to observe any obvious cell morphology changes. Cell cycle regulation is involved in a large array of intracellular differentiation events and several researches established that G0/G1 cell cycle arrest is related to differentiation [56]. The p53 pathway is known to be an important regulatory factor of G0/G1 arrest, and it was regulated by TCQA in treated-hAECs (Fig. 3b). As reported previously, TCQA induces cell cycle arrest and differentiation of hNSCs into three kinds of cells; neuron, oligodendrocyte, and astrocyte [29]. Interestingly, melanocyte precursors are originally derived from the neural crest cells; thus, our current study suggests that TCQA increased G0/G1 arrest by negatively regulating G1/S transition via changing expressions of various genes and moving hAECs toward more lineage-committed cells like neural and pigment cells.

It is important to note that cell cycle can regulate neurogenesis as this process involves proliferation and differentiation [57]. TCQA upregulated the GO of neurogenesis and Notch pathway-related genes (Fig. 3a, b). Notch signaling is essential during embryonic developmental periods of brain, and to maintain the balance between quiescence and active neurogenesis of neural stem cells [58]. The current data showed as well a stimulation of BMP, Transforming Growth Factor Beta (TGF-β), and FGF signaling pathways (Fig. 3). It is widely known that BMP signaling pathway is involved in neural fate commitment, stem cell differentiation, and neurogenesis promotion [59]. On the other hand, the role of TGF-β family in multiple aspects of the nervous system development and function is well established. During adulthood, TGF-β signaling modulates inflammatory responses and plays a protective role against neurodegenerative diseases [60]. These results correlated with the downregulation of inflammation-related genes in TCQA-treated hAECs compared with D7 control (Fig. 3d, e). Next, the highly affected genes by TCQA, whether up or downregulated, are clustered in a heat map showing the differentiation regulation, anti-inflammatory, ATP stimulation, and cell cycle arrest activities of this compound; then some of these genes were validated including *CTNNB1*, *MC1R, BMP5, DMKN, p21, VCAN, IL6*, and *TNFα* (Fig. 4a, b, and Tables 1 and 2). The role of β-catenin in the regulation of pigment and neural stem cells differentiation is previously explained in this study, as for MC1R is established as the main factor dictating pigmentation and melanocytes differentiation [61, 62]. DMKN was identified as one of the most highly expressed genes in keratinocytes, another type of pigment cell, and is involved in their differentiation [63]. VCAN is expressed in human fibroblast and the Extracellular Matrix (ECM) and reported to induce neuronal differentiation and promote neurite outgrowth [64]. For the cell cycle arrest activity, the gene expression of p21 was checked, as we found that p53 pathway was stimulated by TCQA (Figs. 3 and 4, and Table 1). Phosphorylated p53 activates p21, leading to the inhibition of G1/S transition and promoting cell cycle arrest [65]. BMP5 regulates neural crest cell survival, proliferation, and differentiation and promotes dendritic growth [66, 67]. For the inflammatory genes, *IL6* expression was checked because it is an important activator of inflammation and directs the transition from innate to acquired immunity, and is related to pathological situations [68]. Another pro-inflammatory cytokine is TNFα known to interact with IL6 and is associated with neuro-inflammatory response that is linked with several neurological disorders [69]. To look for possible protein interaction between the previously clustered genes, software STRING was used and revealed a potential protein interaction between these genes (Fig. 4c).

Putting together, this study showed that TCQA induced the differentiation of hAECs toward pigment and neural cell lineage by upregulating Wnt, BMP, FGF, and TGF-β signaling pathways. The downregulation of cell cycle-related genes and the upregulation of cell cycle arrest genes contributed to further enhance the differentiation potential of TCQA. Furthermore, TCQA was found to have antioxidant, anti-inflammatory, and ATP stimulating activities. However, further in-depth investigations on the effects of TCQA treatment on morphology, physiology, and protein expression pattern of hAECs are needed to confirm its differentiation effect.

## Supplementary Information

**Additional file 1: Fig. S1.** Comparison gene profiling between D7 TCQA-treated vs D0 control hAECs and D7 TCQA-treated vs D7 control hAECs. A) Venn diagram showing common and unique upregulated sets of DEGs between each exposure. Blue circles denote DEGs between D7 TCQA-treated vs D0 control hAECs and red circles denote DEGs between D7 TCQA-treated vs D7 control hAECs. B) GO analysis of the common upregulated genes set between the two comparison sets. C) Venn diagram showing common and unique downregulated sets of DEGs between D7 TCQA-treated vs D0 control hAECs and D7 TCQA-treated vs D7 control hAECs. D) GO analysis of the common downregulated genes set between the two comparison sets.

## Data Availability

The data that support the findings of this study are available within the paper. The microarray data have been deposited to NCBI, GEO database (accession: GSE153617).

## Abbreviations

HAECs: Human Amnion Epithelial Cells
TCQA: 3,4,5-Tri-*O*-Caffeoylquinic Acid
ESCs: Embryonic Stem Cells
MSCs: Mesenchymal Stem Cells
HAMSCs: Human Amnion Mesenchymal Stromal Cells
CQA: Caffeoylquinic Acid
ATP: Adenosine Triphosphate
SAMP8: Senescence-Accelerated Prone Eight
HNSC: Human Neural Stem cells
BMP: Bone Morphogenetic Protein
CMF-HBSS: Hank’s Basic Salt Solution Calcium and Magnesium Free
EGTA: Ethylene Glycol-bis β-Aminoethyl Ether
DMEM: Dulbecco’s Modified Eagle Medium
FBS: Fetal Bovine Serum
PBS: Phosphate Buffered Saline
RT: Room Temperature
RMA: Robust Multichip Average
TAC: Transcriptome Analysis Console
DAVID: Database of Annotation, Visualization, and Integrated Discovery
GSEA: Gene Set Enrichment Analysis
MC1R: MelanoCortin 1 Receptor
DMKN: Dermokine
p21: Cyclin-Dependent Kinase Inhibitor
VCAN: Versican
IL6: Interleukin 6
TNFα: Tumor Necrosis Factors Alpha
SD: Standard Deviation
DEGs: Differentially expressed genes
GO: Gene Ontology
FGF: Fibroblast Growth Factor
Shh: Sonic Hedgehog
MAPK: Mitogen-Activated Protein Kinase
P53: Tumor Protein p53
TCA: Tricarboxylic Acid Cycle
IFNγ: Interferon Gamma
IL8: Interleukin 8
TGF-β: Transforming Growth Factor Beta
ECM: Extracellular Matrix
