# Supplementary material for: Regulating cell fate of human amnion epithelial cells using natural compounds: an example of enhanced neural and pigment differentiation by 3,4,5-tri-O-caffeoylquinic acid
Source: Cell Commun Signal. 2021 Feb 24;19:26. doi: 10.1186/s12964-020-00697-5 (PMC7903623; doi:10.1186/s12964-020-00697-5)
Supplement: Supplementary file 2 — Additional file 1: Fig. S1. Comparison gene profiling between D7 TCQA-treated vs D0 control hAECs and D7 TCQA-treated vs D7 control hAECs. A) Venn diagram showing common and unique upregulated sets of DEGs between each exposure. Blue circles denote DEGs between D7 TCQA-treated vs D0 control hAECs and red circles denote DEGs between D7 TCQA-treated vs D7 control hAECs. B) GO analysis of the common upregulated genes set between the two comparison sets. C) Venn diagram showing common and unique downregulated sets of DEGs between D7 TCQA-treated vs D0 control hAECs and D7 TCQA-treated vs D7 control hAECs. D) GO analysis of the common downregulated genes set between the two comparison sets. [file 12964_2020_697_MOESM2_ESM.pptx]

## Slide 1
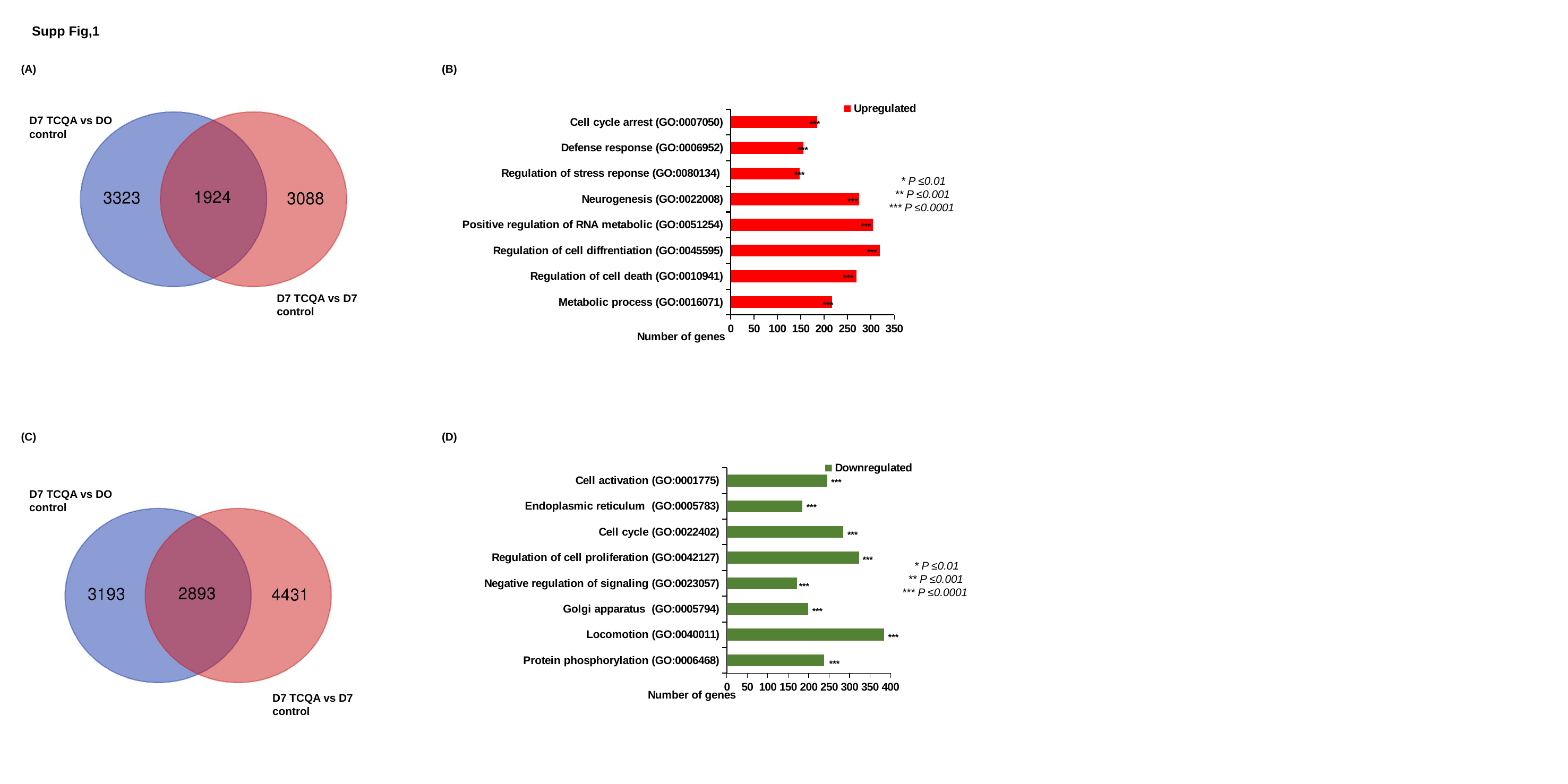

Supp Fig,1
(A) (B)
D7 TCQA vs DO control
D7 TCQA vs D7 control
### Chart
| Category | |
|---|---|
| Metabolic process (GO:0016071) | 217.0 |
| Regulation of cell death (GO:0010941) | 269.0 |
| Regulation of cell diffrentiation (GO:0045595) | 319.0 |
| Positive regulation of RNA metabolic (GO:0051254) | 305.0 |
| Neurogenesis (GO:0022008) | 275.0 |
| Regulation of stress reponse (GO:0080134) | 148.0 |
| Defense response (GO:0006952) | 156.0 |
| Cell cycle arrest (GO:0007050) | 185.0 |***
***
***
***
***
***
***
***
 * P ≤0.01
 ** P ≤0.001
*** P ≤0.0001
(C) (D)
### Chart
| Category | |
|---|---|
| Protein phosphorylation (GO:0006468) | 238.0 |
| Locomotion (GO:0040011) | 385.0 |
| Golgi apparatus (GO:0005794) | 199.0 |
| Negative regulation of signaling (GO:0023057) | 171.0 |
| Regulation of cell proliferation (GO:0042127) | 324.0 |
| Cell cycle (GO:0022402) | 285.0 |
| Endoplasmic reticulum (GO:0005783) | 185.0 |
| Cell activation (GO:0001775) | 245.0 |***
D7 TCQA vs DO control
***
***
***
 * P ≤0.01
 ** P ≤0.001
*** P ≤0.0001
***
***
***
***
D7 TCQA vs D7 control
